# Medical Jurisprudence

**Published:** 1855-09

**Authors:** 


### MEDICAL JURISPRUDENCE.

*Prolonged Retention of Life by Infants who have not Breathed.*—The long period during which life may, under certain circumstances, be retained by the infant which has never breathed, is a fact full of interest to the physiologist, the medical jurist, and the accoucheur. The experiments of Legallois show that, in the mammalia, the fœtus which has not breathed can resist death from submersion much longer than the fœtus in which respiration has been carried on. Puppies and kittens, immediately after birth, may be kept under water for twenty-eight minutes with impunity; when five days old, they perish after sixteen minutes' submersion; and, when fifteen days old, they die as rapidly as other warm-blooded animals of any age, from deprivation of air. The human stillborn fœtus can probably live longer without respiration than any other mammalian fœtus. The following cases are collected in the *Gazette Hebdomadaire* for December 1, 1854, from different sources. They are very striking, and very suggestive to the practical accoucheur.

*Case* 1.—A woman, aged 25, who had tried to conceal her pregnancy, was delivered when seated on a tub. The infant, born without any signs of life, was buried in a sand pit, and, after remaining there for half an hour, was removed, and lived. This case is described by Dr. Weese in 1845, in *Badisch. Ann. f. Staatsarz*, x, 2.

*Case* 2.—In 1850, a young woman was tried by the tribunals of Berlin, who had buried her new-born male infant, believing it to be dead. After one hour, the infant was disinterred, and recalled to life.

*Case* 3.—T. P., a servant, aged 23, was delivered in a stable, when leaning against the wall, alone, and in a state of unconsciousness, about half-past 4 A. M., on the 16th of October. When she came to herself, she found the infant on the ground, having a spade lying upon it, with its cutting edge turned to the body. She took the infant, which was perfectly cold, believing it to be dead, and, with the placenta attached, wrapped it up in her apron, and buried it in the garden. Suspicions arose that she had been confined: she confessed; and at half-past nine the infant was dug up from a depth of thirty *centimetres.* It was found lying on its face, with the placenta under the abdomen. Though cold, apparently dead, and pulseless, the cord was tied. For two hours, P., a surgeon, used means to reanimate it, when at last it began to breathe feebly, gradually signs of life became more evident, and it cried. Some slight wounds were observed on its body: wounds in the neck, which did not bleed at first, bled when the infant was restored. It took the breast greedily. On the 17th and 18th the wounds suppurated; and on the 19th it died of convulsions. The physicians intrusted with the judicial autopsy reported that it had been inhumed before it had breathed; and that it had not breathed till after it was exhumed, and that the statement of the mother was possibly true. She was, therefore, acquitted of the charge of infanticide, but was found guilty of concealment of pregnancy. This case is reported by Dr. Maschka.

*Case* 4.—A. gave birth about noon, in a private house, to an infant, who gave no signs of life. For an hour different unsuccessful attempts were made to animate it. The skin became blue, and gradually warmth left it. It was considered dead, and in three hours it was removed to a cold room. [This occurred in January, and the weather was very cold.] Towards evening it was closed up in a coffin. During the night, the windows of the room remained open. At 11 A. M. on the following day, that is twenty-three hours after the birth, Dr. Maschka, being accidentally at the house, was asked to look at the body. It was perfectly cold and blue; the eyes and mouth were shut; the joints and the extremities were flexible; there was neither rigidity nor cadaveric discoloration. Astonished at this latter circumstance, but not really doubting that the death of the infant was real, Dr. Maschka placed the stethoscope upon the region of the heart, when to his amazement he distinctly heard the sounds of the heart; they were feeble and at long intervals. It was impossible to appreciate the impulse of the heart against the walls of the chest, or to perceive any movement in the corresponding intercostal space. Attempts at resuscitation were made, but without any effect. At the autopsy on the following day cadaveric discolorations and rigidity were present. The lungs were of a deep red, and contained no air; they were heavier than water. There was blood in the left, but none in the right side of the heart. It must be admitted, provided the observation of the reporter be correct, that this infant lived twenty-three hours after birth and never breathed.

*Case* 5.—The following case is extracted from the *Gazette des Tribunaux* of Februaay 20, 1850: A woman was tried for attempting infanticide; she had buried her infant, but it was dug up, breathed, and lived. The following are the facts: Marie and Renee lived with their father at Vernantes, in the *arrondissement* of Bauge (Maine-et-Loire.) On the 16th of May, 1849, Marie was alone in the house with her father. About half-past P. M., Renee came home, found Marie in a swoon, and called the neighbors to her assistance. Marie soon regained consciousness. One of the female neighbors, having observed numerous spots of blood, asked Marie if she had been confined. She replied, "No, it is not time yet." Her father, however, having observed the earth disturbed in a place in the garden, asked her to explain it, when she replied, "I have been confined, but as my child was stillborn, I buried it in the garden." The child was then disinterred. It was found five *centimetres* [about two inches] below the sarface, with its face downwards, and with the placenta attached. Means were used to restore the infant, and it was ere long recalled to life. It was calculated that the infant had been three-quarters of an hour under the ground. The accused was acquitted.

The elaborate memoir of Dr. Maschka terminates with the following summary:

1. New-born infants can live without breathing, not merely for half an hour or an hour, but for a very considerably longer period, even in circumstances the most unfavorable.

2. In such cases there is obviously not only on arrest of blood in the capillaries of the skin, but the vessels of the different organs are either in a state of permanent contraction, or are filled with a column of stagnant blood.

3. The movements of the heart must gradually become very slow. Here a very important question presents itself. How can there be circulation in the infant without respiration, and therefore without the production of arterial blood? This condition of passive life can only exist when assimilation is at a minimum, and when the oxygen of the maternal blood is consumed very slowly. The maternal blood must in such cases be sufficient to maintain life.

Dr. Maschka's essay is contained in the *Vierteljahrschrift f. Hoprakt. Heilkunde*, t. iii, 1854. It contains interesting physiological discussions, which will repay perusal.—*Assoc. Med. Journ.* Dec. 8, 1854.

